# Non-sterile Submerged Fermentation of Fibrinolytic Enzyme by Marine *Bacillus subtilis* Harboring Antibacterial Activity With Starvation Strategy

**DOI:** 10.3389/fmicb.2019.01025

**Published:** 2019-05-17

**Authors:** Shihan Pan, Guiguang Chen, Rui Wu, Xiaoyan Cao, Zhiqun Liang

**Affiliations:** State Key Laboratory for Conservation and Utilization of Subtropical Agro-Bioresources, Guangxi Microorganism and Enzyme Research Center of Engineering Technology, College of Life Science and Technology, Guangxi University, Nanning, China

**Keywords:** *Bacillus subtilis*, fibrinolytic enzyme, non-sterile fermentation, starvation strategy, antibacterial activity

## Abstract

Microbial fibrinolytic enzyme is a promising candidate for thrombolytic therapy. Non-sterile production of fibrinolytic enzyme by marine *Bacillus subtilis* D21-8 under submerged fermentation was realized at a mild temperature of 34°C, using a unique combination of starvation strategy and self-production of antibacterial agents. A medium composed of 18.5 g/L glucose, 6.3 g/L yeast extract, 7.9 g/L tryptone, and 5 g/L NaCl was achieved by conventional and statistical methods. Results showed efficient synthesis of fibrinolytic enzyme and antibacterial compounds required the presence of both yeast extract and tryptone in the medium. At shake-flask level, the non-sterile optimized medium resulted in higher productivity of fibrinolytic enzyme than the sterile one, with an enhanced yield of 3,129 U/mL and a production cost reduced by 24%. This is the first report dealing with non-sterile submerged fermentation of fibrinolytic enzyme, which may facilitate the development of feasible techniques for non-sterile production of raw materials for the preparation of potential drugs with low operation cost.

## Introduction

Formation of endogenous thrombi in blood vessels leads to cardiovascular diseases which have become the leading cause of morbidity and mortality worldwide (Mozaffarian et al., 2015; Seo et al., 2018). Fibrinolytic enzyme catalyzes the breakdown of the fibrin mesh of thrombi, thereby playing an important role in thrombolytic therapy (Kotb, 2013). For decades, the high expenses and undesired side effects caused by clinical use of traditional thrombolytic agents have prompted researchers toward isolating fibrinolytic enzymes from various sources such as snakes, earthworms, insects, plants, algae, and microorganisms (Peng et al., 2005; Kotb, 2014; Silva et al., 2018). Notably, fibrinolytic enzymes originated from microbes have attracted much more medical interest because of relatively high fibrin specificity and low production cost (Cai et al., 2017; Silva et al., 2018).

The commercial availability of microbial fibrinolytic enzyme requires enhanced productivity at an economical viable cost (Avhad and Rathod, 2014). During the past decades, studies involving cost-effective production of fibrinolytic enzymes primarily focused on optimizing low-cost media under sterile conditions (Dabbagh et al., 2014; Vijayaraghavan et al., 2016). However, sterilization is time-consuming and laborious with high energy costs (Zeng et al., 2018). Compared to sterile fermentation, non-sterile fermentation offers several benefits including elimination of sterility, reduced maintenance requirements, relatively simple bioreactor design, and simplified operation (Chen and Wan, 2017), which can significantly reduce fermentation costs. Nevertheless, there is no research concerning fibrinolytic enzyme production by non-sterile submerged fermentation.

Biological control is of vital importance to the success of non-sterile fermentation. A variety of strategies such as starvation strategy, substrate and product inhibition, antimicrobial agents, salts, pH, temperature, inoculation size, immobilization, mixed culture and metabolic engineering were investigated to avoid microbial contamination (Chen and Wan, 2017). Among various strategies for non-sterile fermentation, starvation strategy and antimicrobial agents are proposed for mild operation conditions and efficient inhibition of microbial contaminants during a long fermentation period. The purpose of starvation strategy is to selectively culture individual strains by controlling nutrient levels (Wang et al., 2006; Chen and Wan, 2017). Nutrient deficiency can inhibit cell growth of contaminants but favor the accumulation of target metabolites by some microbes (Chen and Wan, 2017). Antimicrobial agents such as antibiotics, bacteriocins, and essential oils have been applied for food fermentation and preservation (Chen and Wan, 2017; Mathur et al., 2017).

Generally, non-sterile fermentation is implemented with one kind of strategy. The combined use of strategies is also appreciated. For example, the self-production of antimicrobial compounds coupled to efficient growth at low temperature and pH enabled microbial lipid production under non-sterile conditions (Santamauro et al., 2014). Recently, non-sterile lipid fermentation has been realized under a two-stage culture process directed by starvation strategy using a nutrient-limited medium containing *N*-acetylglucosamine with a proper inoculation size (Tang et al., 2018).

In the present study, the efficacy of non-sterile submerged fermentation of fibrinolytic enzyme was evaluated for the first time. Marine *Bacillus subtilis* D21-8, a mutant derived from *B. subtilis* HQS-3 (Huang et al., 2013), was used to produce fibrinolytic enzyme with the aid of starvation strategy under non-sterile conditions. The antibacterial properties of the strain D21-8 were also characterized to elucidate the non-sterile processes. Subsequently, medium optimization was conducted in non-sterile fermentation using traditional and statistical methods. At last, the sterile and non-sterile fermentation processes using the optimized medium were compared. The strategies applied in non-sterile submerged fermentation of fibrinolytic enzyme by the generally regarded as safe (GRAS) strain, *B. subtilis* D21-8, can possibly be exploited beneficially in the cost-effective non-sterile production of health-related products for prevention and treatment of thrombosis.

## Materials and Methods

### Materials

Bovine fibrinogen was purchased from Sigma (St. Louis, MO, United States). Thrombin was provided by Hunan Yige Pharmaceutical Co. Ltd. (Xiangtan, China). Yeast extract (YE) and tryptone were acquired from local suppliers. Other chemicals were of analytical grade.

### Media

Agar slant for strain maintenance was composed of (g/L) 5 glucose, 5 YE, 10 tryptone, 5 NaCl and 20 agar (pH 7.0). Seed medium had the same components as agar slant without adding agar. The basal medium was comprised of (g/L) 10 glucose, 5 YE, 10 tryptone, and 5 NaCl (pH 7.0). Luria Agar, YEPD Agar, and Czapek Agar (ATCC Medium 312) described by Atlas (2010) were applied in antimicrobial assay for cultivation of bacteria, yeasts, and molds, respectively. Media used in this work were prepared with deionized water.

### Microorganisms and Culture Conditions

*Escherichia coli* DH5α, *Lactobacillus* sp. PL17, *Pichia pastoris* GS115, *Saccharomyces cerevisiae* GXJ-1 (Chen et al., 2011), and *Aspergillus niger* GS3-3 (Li et al., 2014) preserved in our lab were used as indicator strains for antimicrobial assay.

*Bacillus subtilis* D21-8 was attained by UV mutagenesis of *B. subtilis* HQS-3 (Huang et al., 2013) with improved fibrinolytic activity. The mutant strain D21-8 was maintained at 4°C on agar slant. The culture conditions of the strain D21-8 were based on our preliminary experiments at shake-flask level, which indicated the tested strain grew well in the seed medium (37°C, 160 rpm) as well as in the basal medium (34°C, 220 rpm). One loop of cells of the strain was transferred to a 250 mL Erlenmeyer flask containing 50 mL of seed medium. The flask was incubated at 37°C and 160 rpm in a constant temperature shaker (SKY-2112B; SUKUN Industry & Commerce Co. Ltd., Shanghai, China) for 16 h to prepare inoculum. All fermentations were conducted in a 250 mL shake flask with 50 mL of medium. Inoculum size, temperature, and shaking speed were defined as 2% (v/v), 34°C, and 220 rpm, respectively.

### Influences of Starvation Strategy and Self-Production of Antibacterial Substances

The starvation strategy applied in this study was developed from the method described by Lin et al. (2011). To create a nutrient-deficient environment dominated by *B. subtilis* D21-8, the basal medium was formulated with the same type of nutrients varying in concentration as compared to the seed medium. Our preliminary experiments showed that glucose present in the basal medium was rapidly consumed after inoculation of the strain D21-8, which was responsible for the establishment of a carbon-deficient environment. For evaluating the effects of starvation strategy on contaminant control, we performed non-sterile submerged fermentation using the basal medium. The non-sterile basal media inoculated with and without *B. subtilis* D21-8, respectively, were incubated for 30 h. Samples were withdrawn at different intervals for analysis. Time courses of DCW and glucose concentration were compared between non-sterile fermentations with and without inoculation of *B. subtilis* D21-8, respectively. Fibrinolytic activity and antimicrobial activity of the culture supernatants of the strain D21-8 were also determined.

### Medium Optimization in Non-sterile Fermentation

After verifying the efficacy of bacterial contaminant control by starvation strategy and self-production of antibacterial compounds by *B. subtilis* D21-8, medium optimization was performed under non-sterile conditions. NaCl was fixed at 5 g/L in non-sterile media and samples were harvested after 48 h of fermentation.

Medium components were preliminarily optimized by the one-factor-at-a-time method using non-sterile media. Different concentrations of glucose (5–100 g/L), YE (0–50 g/L), and tryptone (0–50 g/L) were successively investigated to improve fibrinolytic activity. Effects of inorganic salts (2 g/L) including Na_2_HPO_4_ ⋅ 12H_2_O, KH_2_PO_4_, MgSO_4_ ⋅ 7H_2_O, and CaCl_2_ on fibrinolytic activity were evaluated as well.

Further experiments designed with response surface methodology (RSM) (Zhu et al., 2014) were carried out to explore the relationships between the key factors influencing fibrinolytic enzyme production. A Box-Behnken Design (BBD) (Box and Behnken, 1960) was performed. Table 1 shows the experimental design with variables being set at three levels (−1, 0, and 1); −1, 0, and 1 represent the low, moderate, and high levels of the factors, respectively. RSM was implemented to optimize the concentrations of variables and to elucidate the interaction effects of these factors on fibrinolytic activity. The experimental results were fitted with the following second order polynomial equation:

**Table 1:** Design and results of BBD.

Run no.	Factors (g/L)			Fibrinolytic activity	DCW
	Glucose	YE	Tryptone	(U/mL)	(g/L)
1	30 (1)	5 (0)	5 (−1)	1878	2.36
2	30 (1)	5 (0)	15 (1)	2041	3.17
3	10 (−1)	5 (0)	15 (1)	2375	2.50
4	20 (0)	5 (0)	10 (0)	2995	2.66
5	20 (0)	5 (0)	10 (0)	2965	2.65
6	20 (0)	5 (0)	10 (0)	2904	2.66
7	20 (0)	2 (−1)	5 (−1)	700	2.27
8	20 (0)	2 (−1)	15 (1)	2317	3.19
9	20 (0)	5 (0)	10 (0)	2934	2.67
10	30 (1)	2 (−1)	10 (0)	942	2.77
11	20 (0)	5 (0)	10 (0)	2907	2.64
12	10 (−1)	8 (1)	10 (0)	2192	4.81
13	10 (−1)	5 (0)	5 (−1)	2103	2.23
14	10 (−1)	2 (−1)	10 (0)	1764	2.63
15	20 (0)	8 (1)	15 (1)	1407	5.13
16	30 (1)	8 (1)	10 (0)	1891	3.35
17	20 (0)	8 (1)	5 (−1)	2955	2.61

*Numbers in parentheses represent coded levels. The optimal concentrations of the factors obtained by the one-variable-at-a-time approach were defined as the moderate levels of 0.*

(1) $$y={\text{β}}_{0}+\sum_{\text{i}=1}^{\text{k}}{\text{β}}_{\text{i}}x_{\text{i}}+\sum_{\text{i}=1}^{\text{k}}{\text{β}}_{\text{ii}}x_{\text{i}}^{2}+\sum_{\text{i}=1}^{\text{k}-1}\sum_{\text{j}=2}^{\text{k}}{\text{β}}_{\text{ij}}x_{\text{i}}x_{\text{j}}$$

where *y*: response (fibrinolytic activity); *x*_i_ and *x*_j_: independent variables; *k*: the number of variables; β_0_: constant; and β_i_, β_ii_, and β_ij_: coefficients for the linear, quadratic, and interaction effects, respectively. Three parallel experiments using the optimal medium were conducted for validation of the model.

### Batch Fermentation Under Sterile and Non-sterile Conditions

The statistically optimized medium sterilized at 115°C for 30 min (initial pH 6.9) and the non-sterile one (initial pH 6.8) were used in batch fermentation by *B. subtilis* D21-8 under sterile and non-sterile conditions, respectively. Experiments were carried out at 34°C and 220 rpm in a 250 mL shake flask supplemented with 50 mL of the optimized medium and 1 mL of inoculum. All fermentations lasted for 72 h and cultures were periodically taken for pH monitoring as well as analysis of biomass, glucose, fibrinolytic activity, and antibacterial activity.

### Assays

Fibrinolytic activity was determined as described by Astrup and Mullertz (1952) using urokinase as a control. Antimicrobial activity was assayed by the agar well diffusion method (Kabore et al., 2013) with culturing the indicator strains at appropriate temperatures and using ampicillin (100 μg/mL) as reference. Cell growth was measured by dry cell weight (DCW). Thirty mL samples were centrifuged at 10,000 × g at 4°C for 10 min. The precipitates were washed twice with deionized water and dried in a hot air oven (HJJF-71; Yuejin Medical Instruments Co., Ltd., Shanghai, China) at 90°C to a constant weight for measurement. Contaminants (bacteria) were evaluated on Luria Bertani (LB) agar plates spread with serially diluted culture broth. Glucose content was quantified by an oxidase electrode biosensor (SBA-40D; Shandong Academy of Sciences, Jinan, China).

### Data Analysis

Three parallel replicates were employed in all experiments. Results were statistically analyzed by analysis of variance (ANOVA) and multiple comparisons using Minitab 16 software (Minitab Inc., United States). Design-Expert 8.0.6 software (Stat-Ease Inc., United States) was used for experimental design and regression analysis.

## Results

### Contaminant Control by Starvation Strategy and Self-Production of Antibacterial Substances

**FIGURE 1:**
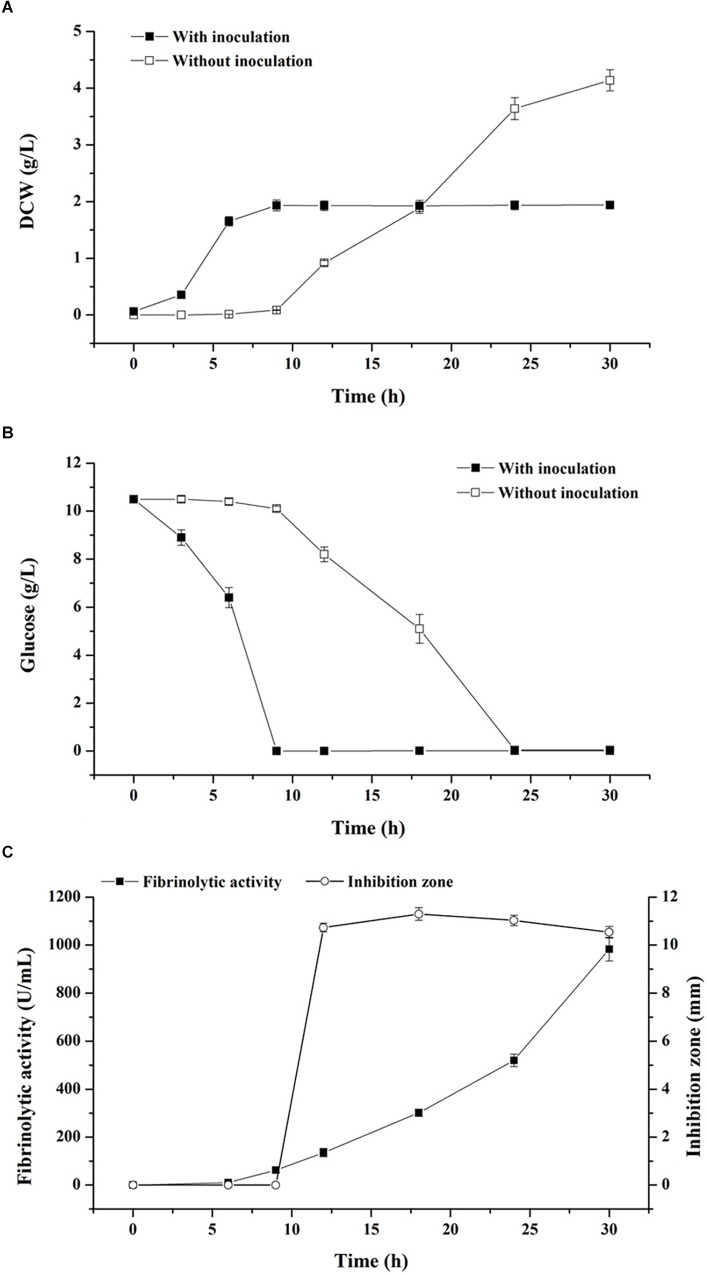
Effects of starvation strategy and self-production of antibacterial compounds on contaminant control and fibrinolytic enzyme production. Cell growth **(A)** and glucose consumption **(B)** during fermentation using the non-sterile basal medium with and without inoculation of *B. subtilis* D21-8. **(C)** Time courses of fibrinolytic activity and antibacterial activity against *E. coli* DH5α in non-sterile fermentation by *B. subtilis* D21-8 using the basal medium. Antibacterial activity was indicated by the diameter of the inhibition zone.

The basal medium was employed to evaluate the effect of starvation strategy aimed at creating a nutrient-deficient condition for biological control (Figure 1). As illustrated in Figure 1A, when the exponential-phase seed cultures were inoculated into the basal medium, nutritional conditions before and after inoculation were similar for cellular metabolism, thus boosting nutrient assimilation as well as reducing the length of the lag phase of *B. subtilis* D21-8 to less than 3 h. The maximum DCW of 1.93 g/L was obtained at 9 h with inoculation of the strain D21-8 while DCW that was observed in the basal medium without inoculation was 0.09 g/L at the same time. It was evident that at a mild temperature of 34°C, bacterial contaminants experienced a much longer lag phase (9 h) in the basal medium than the strain D21-8. As a result, the strain gained plenty of time to proliferate and outnumber the contaminants. Meanwhile, glucose exhaustion occurred at stationary phase of the strain D21-8, inhibiting biomass accumulation of the contaminants (Figure 1B). In addition, contaminants were not detected on LB agar plates during the stationary phase of *B. subtilis* D21-8 (9–30 h) (Figure 1A). Consequently, glucose could be considered as the starvation factor in the production of fibrinolytic enzyme during stationary phase.

Antimicrobial assays showed *in situ* antibacterial activity against *Escherichia coli* DH5α appeared at 12 h with an inhibition zone of 10.73 mm and then the inhibition zone grew up to 11.30 mm at 18 h but slowly diminished thereafter (Figure 1C). On the other hand, fibrinolytic activity persistently increased from 9 to 30 h. The distinct synthetic patterns between fibrinolytic enzyme and antimicrobial compounds during the stationary phase implied they are distinguished secondary metabolites. It was then confirmed by the fact that the purified fibrinolytic enzyme was not active against *E. coli* DH5α (data not shown).

**FIGURE 2:**
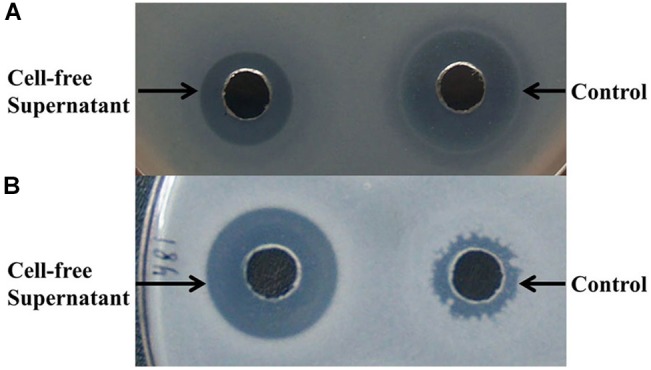
Assay of antibacterial activity against *E. coli* DH5α **(A)** and *Lactobacillus* sp. PL17 **(B)**. **(A,B)** 50 μL of samples were added into the wells; cell-free supernatant was obtained after 18 h of fermentation by *B. subtilis* D21-8 using the non-sterile basal medium; control: 100 μg/mL ampicillin.

To further determine the inhibitory spectrum of the antimicrobial compounds produced by *B. subtilis* D21-8, antimicrobial assays were carried out. The cell-free supernatant obtained after 18 h of incubation exhibited a lower inhibitory effect against *E. coli* DH5α (11.30 mm) than ampicillin (14.79 mm) (Figure 2A) but resulted in a significantly bigger inhibition zone against *Lactobacillus* sp. PL17 (14.13 mm) than the control (9.24 mm) (Figure 2B). For the positive control (ampicillin), the inhibition zones against bacteria with dimensions of <9.0, 9.0–13.5, and >13.5 mm were considered resistant, medium sensitive and sensitive, respectively. Thus, the antibacterial substances synthesized by the strain D21-8 showed sensitive and medium-sensitive effects against *Lactobacillus* sp. PL17 and *E. coli* DH5α, respectively. However, the tested fungi were resistant to the cell-free supernatant (data not shown).

Although the antimicrobials produced by *B. subtilis* D21-8 could not inhibit the tested fungi, results showed very few fungi (mainly *Aspergillus niger*) were present in the non-sterile basal medium when incubated without inoculation of the strain D21-8, whereas fast-growing bacteria such as *E. coli* dominated in the medium, which suggested *in situ* antibacterial activity against Gram-negative *E. coli* DH5α (medium sensitive) and Gram-positive *Lactobacillus* sp. PL17 (sensitive) was promising for bacterial contaminant control.

### Effects of Medium Components on Fibrinolytic Activity

**FIGURE 3:**
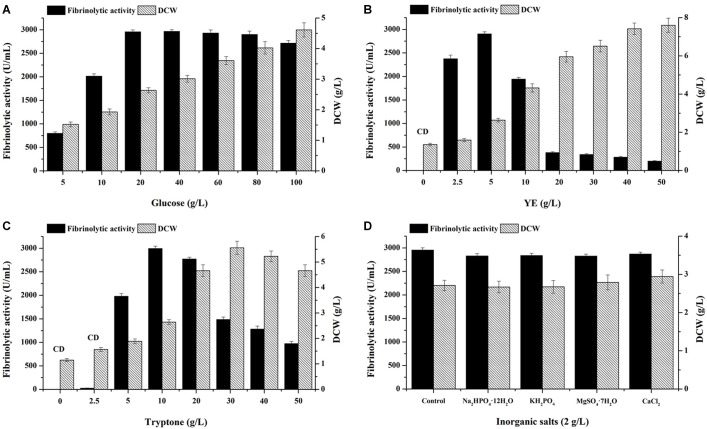
Effects of glucose **(A)**, YE **(B)**, tryptone **(C)**, and inorganic salts **(D)** on fibrinolytic activity. **(B,C)** CD, Contaminants were dominated on LB agar plates inoculated with serially diluted culture broth harvested at 48 h. **(D)** Control (g/L): 20 glucose, 5 YE, 10 tryptone and 5 NaCl.

The concentrations of medium components including glucose, YE and tryptone were preliminarily optimized by single factor experiments using non-sterile media. Glucose concentration was first evaluated by fixing YE and tryptone at 5 and 10 g/L, respectively (Figure 3A). With glucose content increasing, biomass gradually increased. The highest fibrinolytic activity was obtained with 20 g/L glucose (2,955 U/mL), which was significantly higher than that using the basal medium containing 10 g/L glucose (2,014 U/mL) (*P* < 0.05). No significant difference in fibrinolytic activity was observed among the treatments with 20, 40, 60, and 80 g/L glucose, respectively. However, further increase of glucose to 100 g/L reduced fibrinolytic activity, suggesting substrate inhibition occurred. Thus, 20 g/L glucose was retained for the next experiments.

As for nitrogen sources, absence of either YE (Figure 3B) or tryptone (Figure 3C) in fermentation medium led to no yield of fibrinolytic enzyme and dominance of bacterial contaminants at the end of fermentation, which suggested both YE and tryptone are indispensable for the co-production of fibrinolytic enzyme and antibacterial compounds by *B. subtilis* D21-8. Besides, relatively low concentrations of tryptone (2.5 g/L) and YE (5 g/L) caused very little production of fibrinolytic enzyme (Figure 3C) with abundance of contaminants. Subsequent results showed 5 g/L YE (Figure 3B) and 10 g/L tryptone (Figure 3C) yielded the highest fibrinolytic activity. Nevertheless, further increase of YE and tryptone exerted significantly negative effects on fibrinolytic enzyme production, probably due to substrate inhibition. In addition, increasing YE facilitated cell growth of the strain D21-8, while tryptone with a concentration higher than 40 g/L hindered biomass accumulation. Similarly, high tryptone concentration significantly inhibited both cell growth and nattokinase production (Cho et al., 2010).

Regarding inorganic salts on fibrinolytic enzyme production, no improvement in fibrinolytic activity (Figure 3D) was found after adding the tested inorganic salts to the control medium, revealing sufficient mineral salts present in YE and tryptone for synthesis of fibrinolytic enzyme. In addition, glucose was completely consumed at the end of fermentation for the control and the four experimental groups (Figure 3D), suggesting similar usage of glucose for cell growth and fibrinolytic enzyme production by the strain D21-8.

### Medium Optimization Using RSM

Based on the above single-factor experiments, 20 g/L glucose, 5 g/L YE, and 10 g/L tryptone were chosen as the zero levels for BBD. Experimental design and the corresponding results were presented in Table 1. The highest fibrinolytic activity (2,904–2,995 U/mL) was observed at the center point (Runs 4, 5, 6, 9, and 11) as well as in Run 17, suggesting 5–8 g/L YE and 5–10 g/L tryptone were among the optimal concentrations for fibrinolytic enzyme production. In contrast, runs 7 and 15 with the lowest and highest levels of nitrogen sources (YE and tryptone), respectively, resulted in low yields of fibrinolytic enzyme. DCW obtained with the highest fibrinolytic activity was around 2.65 g/L and bacterial contamination was not observed on LB agar plates in any of the 17 runs. Assays indicated glucose was exhausted at the end of fermentation (48 h) for all runs (Table 1).

By applying multiple regression analysis on the experimental data, the response (*Y*: fibrinolytic activity) and the tested variables (*A*: glucose; *B*: YE; *C*: tryptone) in coded values were related by the following equation:

(2) $$\begin{array}{c} Y=2921-210.25A+340.25B+63C+130.25\mathrm{AB}-27.25\mathrm{AC}\\ \;-791.25\mathrm{BC}-484.63A^{2}-739.13B^{2}-337.12C^{2} \end{array}$$

**FIGURE 4:**
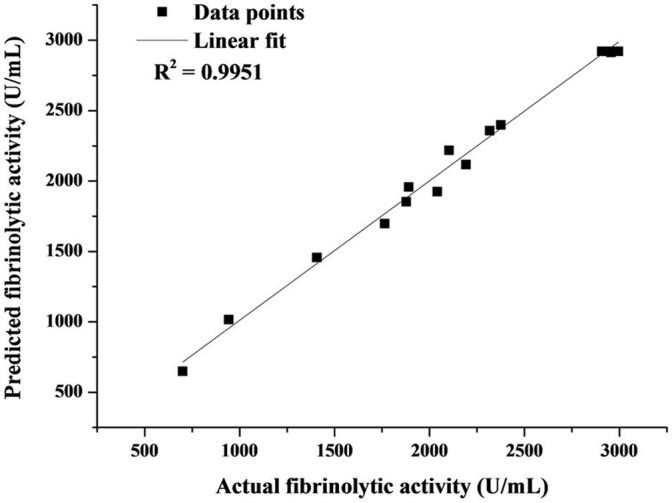
Correlation between experimental and predicted fibrinolytic activity.

The variance analysis of the regression model (Table 2) revealed high values of *R*^2^ (0.9904) and Adj *R*^2^ (0.978) as well as the non-significant lack of fit (*P* = 0.1279), leading to a good correlation between the actual and predicted fibrinolytic activity (Figure 4). Thus, the acquired model was valid and could be adopted in this work. As presented in Table 2, fibrinolytic activity was significantly affected by the linear and interactive effects of glucose and YE, the interactive effects of YE and tryptone, and the quadratic effects of all factors (*P* < 0.05). Nonetheless, the linear effect of tryptone on fibrinolytic activity was not significant (*P* = 0.1355).

**Table 2:** Analysis of variance for the results of BBD.

Source	Sum of squares	df	Mean square	*F*-value	Prob > *F*
Model	8.04E+06	9	8.93E+05	80.06	<0.0001
*A*	3.54E+05	1	3.54E+05	31.7	0.0008
*B*	9.26E+05	1	9.26E+05	83.01	<0.0001
*C*	31752	1	31752	2.85	0.1355
*AB*	67860.25	1	67860.25	6.08	0.0431
*AC*	2970.25	1	2970.25	0.27	0.6218
*BC*	2.50E+06	1	2.50E+06	224.46	<0.0001
*A*^2^	9.89E+05	1	9.89E+05	88.63	<0.0001
*B*^2^	2.30E+06	1	2.30E+06	206.17	<0.0001
*C*^2^	4.79E+05	1	4.79E+05	42.89	0.0003
Residual	78100.5	7	11157.21		
Lack of fit	56634.5	3	18878.17	3.52	0.1279
Pure error	21466	4	5366.5		
Cor total	8.12E+06	16			

**FIGURE 5:**
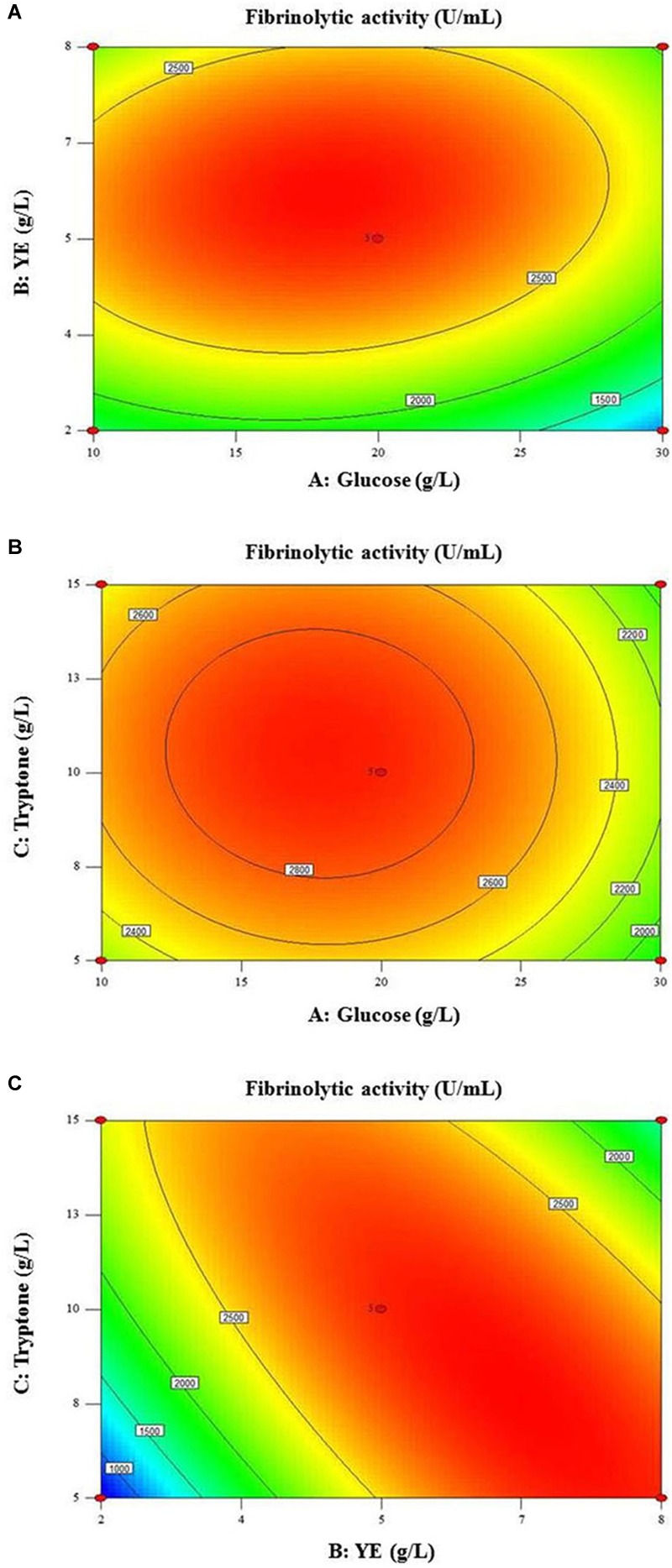
Contour plots showing interactive effects of **(A)** glucose and YE, **(B)** glucose and tryptone and **(C)** YE and tryptone on fibrinolytic activity.

The two-dimensional (2D) response surface plots (Figure 5) display the optimal value of fibrinolytic activity and interactions between the nutritional variables. The smallest ellipse in the contour plot indicates the optimum values of variables at the maximum predicted response (Qing et al., 2018). Significant interaction between glucose and YE on fibrinolytic activity was found with optimal concentrations of glucose and YE ranging 15–20 and 5–7 g/L, respectively (Figure 5A). On the contrary, little interactive effect was observed between glucose and tryptone (Figure 5B). High ellipticity depicted in Figure 5C revealed a strong interactive effect between YE and tryptone for fibrinolytic enzyme production.

As estimated by the model equation, the optimum fibrinolytic activity of 2,998 U/mL exists when concentrations of glucose, YE, and tryptone are 18.5, 6.3, and 7.9 g/L, respectively. The maximum fibrinolytic activity achieved experimentally was found to be 3,129 U/mL using the non-sterile optimized medium, which was close to the predicted result. Consequently, statistical methods using BBD and RSM were successfully applied to study the influence of nutritional variables and to optimize enzyme yield.

### Comparison Between Sterile and Non-sterile Fermentation of Fibrinolytic Enzyme

**FIGURE 6:**
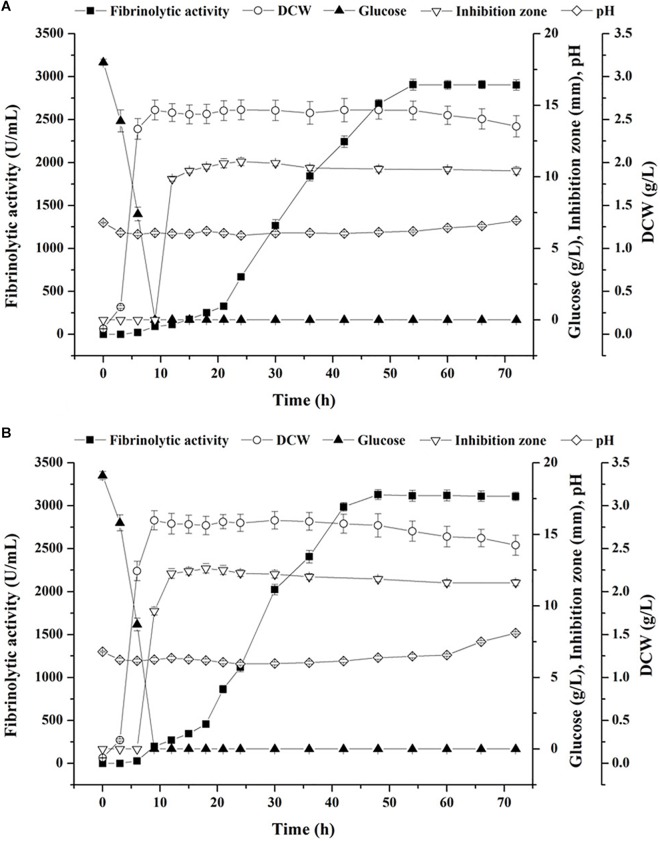
Time courses of batch fermentation of fibrinolytic enzyme with the optimized medium by *B. subtilis* D21-8 under sterile **(A)** and non-sterile **(B)** conditions.

The optimized medium obtained by experiments directed by starvation strategy was applied in submerged batch fermentation under sterile (Figure 6A) and non-sterile (Figure 6B) conditions. A shortened lag phase (<3 h) and a glucose-limited condition at stationary phase were both observed in sterile and non-sterile fermentation (Figure 6). Although the trends of cell growth and glucose consumption were similar under the two conditions, the maximum biomass (2.83 g/L) and the initial glucose content (19.1 g/L) obtained using the non-sterile medium were a little higher than those using the sterile medium (2.61 and 18.0 g/L, respectively). With respect to fibrinolytic enzyme production, the highest fibrinolytic activity achieved under the non-sterile condition (3,129 U/mL at 48 h) was relatively higher than that in sterile fermentation (2,906 U/mL at 54 h) (Figure 6). In addition, after reaching the highest fibrinolytic activity, biomass started to decrease at a low rate under both conditions, which was accompanied with a continuous rise of pH value and which, in turn, implied accumulation of toxic compounds and exhaust of available nutrients at the end of the stationary phase (Erkmen and Bozoglu, 2016).

Aside from fibrinolytic enzyme production, *in situ* antibacterial activity was detected during the stationary phase. As illustrated in Figure 6A, the inhibition zone against *E. coli* DH5α emerged at 12 h in sterile fermentation, expanded up to 11.57 mm at 24 h and shrank slowly afterwards, indicating an inherent nature of biosynthesis of antibacterial substances during the stationary phase of *B. subtilis* D21-8. Notably, antibacterial activity against *E. coli* DH5α was observed at 9 h in non-sterile fermentation and then reached its highest level at 18 h (13.12 mm) (Figure 6B), demonstrating that the production of antibacterial compounds in non-sterile fermentation was apparently faster and higher than that obtained in the sterile medium.

### Benefits of the Non-sterile Fermentation Strategies

Starvation strategy and self-production of antimicrobials by *B. subtilis* D21-8 applied in non-sterile fermentation resulted in a high fibrinolytic enzyme yield and a low operation cost. Regarding fibrinolytic enzyme yield, a variety of analytical methods are used in determination of fibrinolytic activity in the literature, and it is hard to compare the yields assessed by different methods. As a result, only those reports using fibrin plate method that is the same or similar to that proposed by Astrup and Mullertz (1952) for measuring fibrinolytic activity were compared with the present study for the production of fibrinolytic enzyme. As presented in Table 3, data attained in this work showed high levels of fibrinolytic enzyme yield (3,129 U/mL) and productivity (65.2 U/mL/h), which were among the highest using the fibrin plate method. Therefore, the non-sterile optimized medium exhibited great potential for mass-production of fibrinolytic enzyme.

**Table 3:** Comparison of fibrinolytic enzyme production between *B. subtilis* D21-8 and other strains by submerged batch fermentation.

Strains	Medium sterilization	Fermentation time (h)	Fermentation systems	Yield (U/mL)/productivity (U/mL/h)
*B. subtilis* BK-17 (Lee et al., 1999)	Yes	13	5 L fermenter	1100/84.6
*B. subtilis* DC-2 (Ashipala and He, 2008)	Yes	118.77	500 mL flask	1223.61/10.3
*B. subtilis* LD-8547 (Wang et al., 2008)	Yes	72	15 L fermenter	4220/58.6
*Bacillus* sp. UFPEDA 485 (Sales et al., 2013)	Yes	84	250 mL flask	835/9.9
*B. subtilis* I-2 (Bajaj et al., 2014)	Yes	N	Shake flask	797.28/N
*B. cereus* SRM-001 (Narasimhan et al., 2015)	Yes	24	500 mL flask	1450/60.4
*B. subtilis* D21-8 (this study)	No	48	250 mL flask	3129/65.2

*Yield (fibrinolytic activity) was evaluated by the fibrin plate method. N, Not provided.*

For fermentation cost, the sterile and non-sterile fermentation using the optimized medium were compared and the results are shown in Table 4. All the prices of feedstocks and energy were provided by local suppliers. To produce 1 × 10^9^ U fibrinolytic enzyme, the production costs for sterile and non-sterile fermentation were $64.15 and $48.80, respectively, indicating a 24% decrease in fermentation cost when using the non-sterile optimized medium for fibrinolytic enzyme production. In addition, the sterilization cost ($11.16) contributed to approximately 17% of total fermentation cost in sterile fermentation. Nonetheless, the cost for fermentation medium occupied the largest portion of production cost in both fermentations as a result of high prices of YE and tryptone. Future work needs to be focused on replacing YE and tryptone with low-cost nitrogen sources for further reduction in fermentation cost of fibrinolytic enzyme.

**Table 4:** Comparison of cost between sterile and non-sterile fermentation with the optimized medium to produce 1 × 10^9^ U fibrinolytic enzyme.

Parameters	Fermentation types		Unit prices ($)
	Sterile	Non-sterile	
*Fermentation medium*			
Glucose	6.366 kg	5.912 kg	0.558 kg^−1^
YE	2.168 kg	2.013 kg	6.731 kg^−1^
tryptone	2.719 kg	2.525 kg	8.857 kg^−1^
NaCl	1.721 kg	1.598 kg	0.047 kg^−1^
Water	0.344 ton	0.319 ton	0.53 ton^−1^
Cost for fermentation medium	$42.49	$39.46	
*Energy consumption*			
Energy for sterilization (115°C)	137.6 kW ⋅ h	N	0.08 kW^−1^h^−1^
Energy for fermentation (34°C, 220 rpm)	129.6 kW ⋅ h	115.2 kW ⋅ h	0.08 kW^−1^h^−1^
Fermentation time	54 h	48 h	
Fibrinolytic enzyme yield	2,906 U/mL	3,129 U/mL	
Operation cost	$21.66	$9.34	
Total fermentation cost	$64.15	$48.80	

*Unit prices were provided by local suppliers and available in December 2018. N, not adopted.*

## Discussion

Microbial fibrinolytic enzyme, especially from the genus *Bacillus*, could be useful to develop potent thrombolytic agents for the therapy of cardiovascular diseases (Kotb, 2013; Vijayaraghavan et al., 2016). Mass-production of the enzyme with low cost is critical for its commercial success. Compared to traditional fermentation under sterile conditions, non-sterile fermentation eliminates the process of sterilization with high energy consumption, contributing to the economic production of various biochemicals (Santamauro et al., 2014; Tang et al., 2018; Zeng et al., 2018). However, little attention was paid to non-sterile fermentation of fibrinolytic enzyme. For the first time, we managed to realize cost-effective non-sterile production of fibrinolytic enzyme by marine *Bacillus subtilis* D21-8 under submerged fermentation.

To control microbial contaminants in non-sterile submerged fermentation, starvation strategy was introduced in this work. Our preliminary studies showed that, despite various carbon sources and organic nitrogen sources incorporated in the medium, the extracellular fibrinolytic enzyme secreted by *B. subtilis* D21-8 was mainly produced at the stationary phase when nutrients were deficient. To apply starvation strategy for non-sterile fermentation of fibrinolytic enzyme, we wondered if using a non-sterile fermentation medium with a composition close to the seed medium could significantly shorten the lag phase of the strain D21-8, thus enabling the strain to outcompete microbial contaminants and become the dominated species at the stationary phase when starvation strategy takes effect.

Based on the previous research, *B. subtilis* uses glucose as the most preferred source for carbon and energy (Stulke and Hillen, 2000) and YE and tryptone were confirmed as suitable nitrogen sources for nattokinase production by *Bacillus* species (Liu et al., 2005; Cho et al., 2010). Hence, glucose, YE, and tryptone were used to form the seed medium, the basal medium, and the fermentation media with the addition of 5 g/L NaCl to maintain an appropriate osmotic pressure favoring cell growth of the marine *B. subtilis* D21-8. Results showed in the non-sterile basal medium that the strain D21-8 could grow much faster than the contaminants, with a short lag phase of <3h and a glucose-exhausted condition that was observed in the stationary phase (9–30 h) when fibrinolytic enzyme was steadily produced without being compromised by the contaminants. It appears starvation strategy, by means of shortening the lag phase, works—but whether nutrient depletion is the only reason for efficient control of microbial contamination still needs in-depth study.

It is well known that antimicrobial bacteriocins produced by food-grade lactic acid bacteria (LAB) are applied in biocontrol (Erkmen and Bozoglu, 2016). Bacteriocins were also found in several strains of *B. subtilis* (Kabore et al., 2013; Khochamit et al., 2015; Lee and Chang, 2018). Considering the possibility of existence of natural antimicrobials like bacteriocins synthesized by *B. subtilis* D21-8, the cell-free supernatants of the strain were subjected to antimicrobial assays. Assays revealed that the strain D21-8 was able to produce antibacterial substances which significantly inhibited cell growth of the primary contaminants such as *E. coli*.

Dangerous pathogens like *Bacillus anthracis* were not used as indicator strains in the antimicrobial assays because we lack strictly safe protection when handling the pathogens which may cause severe contamination in our lab. Even though *in situ* antibacterial activity against microbial pathogens was not evaluated, contaminants were not detected on LB agar plates during 30 h of non-sterile fermentation using the basal medium inoculated with *B. subtilis* D21-8. It was supposed that the pathogens and other bacterial contaminants were either too few in number to be detectable or thoroughly inhibited by the antibacterial compounds produced by the strain D21-8 as well as the growth-limiting environment created by the adopted starvation strategy.

Accordingly, the combination of starvation strategy and self-production of antibacterial substances contributed to the successful contaminant control in submerged fermentation of fibrinolytic enzyme using the non-sterile basal medium in 250 mL shake flask at a mild temperature of 34°C, which established a solid foundation for medium optimization under non-sterile conditions.

Single factor experiments were carried out to preliminarily optimize the concentrations of medium components, namely glucose, YE and tryptone, for enhancing fibrinolytic activity. It turned out that the absence of YE or tryptone as well as low contents of the two nitrogen sources in the medium led to little fibrinolytic enzyme production and dominance of contaminants, which could be attributed to a lack of precursors for biosynthesis of fibrinolytic enzyme and the antibacterial substances. It was well documented that in *B. subtilis*, glutamine is the preferred nitrogen source and the precursor of all nitrogen-containing compounds (Fisher and Sonenshein, 1991), and high nattokinase production was obtained in the presence of sodium glutamine (Liu et al., 2005). Chen and Chao (2006) reported that glutamine and aspartic acid play a determining role in nattokinase production. Therefore, certain levels of nitrogen sources which provide enough precursors such as glutamine and other amino acids are required for enhanced production of fibrinolytic enzyme. It could be concluded that the production of fibrinolytic enzyme and antibacterial compounds by *B. subtilis* D21-8 was highly dependent on nitrogen sources. A suitable ratio of YE and tryptone in fermentation medium is crucial for enhancing fibrinolytic activity and controlling bacterial contaminants in non-sterile submerged fermentation.

The traditional one-variable-at-a-time approach is simple, but it frequently fails to determine the mutual interaction between the variables and to locate the region of optimum response (Liu et al., 2005; Avhad and Rathod, 2015). Hence, RSM, widely used in the literature for process optimization, was further applied for optimizing the fermentation medium under non-sterile conditions. BBD experiments and the corresponding RSM analysis were successfully conducted, generating a valid regression model for predicting the optimum concentrations of medium components. The regression model indicated fibrinolytic enzyme production was significantly affected by glucose and YE (*P* < 0.001) while tryptone alone showed a non-significant effect. Indeed, tryptone, a pancreatic digest of casein, is commonly used as a source of amino acids (Atlas, 2010), which plays a less important role in fibrinolytic enzyme production than glucose, which is used as the major source of carbon and energy, and YE, which is employed as a source of amino acids and growth factors such as vitamins, fatty acids and trace metals. Regardless of the individual non-significant effect of tryptone, the 2D contour plot demonstrated tryptone strongly interacted with YE for fibrinolytic enzyme production. It could be inferred that YE and tryptone are essential and compensatory to each other for efficient production of fibrinolytic enzyme. Through experimental validation, 18.5 g/L glucose, 6.3 g/L YE, and 7.9 g/L tryptone constituted the optimal medium for non-sterile fermentation of fibrinolytic enzyme by *B. subtilis* D21-8.

After obtaining the optimized fermentation medium by traditional and statistical methods, batch submerged fermentation using the strain D21-8 under sterile and non-sterile conditions was investigated. Results showed non-sterile fermentation resulted in higher biomass and productivity of fibrinolytic enzyme and antimicrobials than sterile fermentation, which demonstrated that the loss and variation of medium components in the sterile medium due to heat treatment exerted negative impacts on the metabolism of the strain. As suggested by Gupta et al. (2002), extracellular protease production manifests nutrient limitation at the onset of stationary phase, and the final protease yield during stationary phase is also determined by the biomass. In this case, the non-sterile medium provided a better nutritional condition for cell growth at the exponential phase and fibrinolytic enzyme production at the stationary phase. It was speculated that YE and tryptone utilized as complex carbon–nitrogen sources in the optimized medium contributed to the slow release of available nutrients for energy formation when glucose, an efficient carbon-energy substrate, was exhausted at the stationary phase. In the meantime, the steady increase of fibrinolytic activity during the stationary phase indicated decomposition of preaccumulated substances produced by the living cells and lysis of the dead cells by multiple proteases to generate essential nutrients for cell survival (Kwon et al., 2011). It could be proposed that the non-sterile optimized medium with intact nutrients performed better than the sterile one not only in fibrinolytic enzyme production but also in synthesis of antibacterial compounds, whose timely generation ensured effective contaminant control throughout 72 h of non-sterile fermentation.

Bacterial contamination is one of the major obstacles to the large-scale production of biochemicals with high yield and productivity because it causes considerable economic losses as a result of bacteria competing for substrates and inhibiting cell growth of the fermentative strain (Tang et al., 2018). Traditional process sterilization would bring excessive costs and sometimes serious contamination could occur, owing to insufficient sterilization of raw materials and equipment. In the present study, the non-sterile optimized medium for fibrinolytic enzyme production was developed based on starvation strategy and efficient self-production of antibacterial compounds for effective control of bacterial contamination, which gained significant technical and economic advantages such as high fibrinolytic enzyme yield and low fermentation cost.

The fibrinolytic enzyme yield of 3,129 U/mL achieved in this work using the non-sterile optimized medium was significantly higher than those of relevant studies using sterile media as reported by Lee et al. (1999) (1,100 U/mL), Ashipala and He (2008) (1,223.61 U/mL), Sales et al. (2013) (835 U/mL), Bajaj et al. (2014) (797.28 U/mL), and Narasimhan et al. (2015) (1,450 U/mL). Wang et al. (2008) reported higher fibrinolytic activity of 4,220 U/mL but lower productivity of 58.6 U/mL/h when compared to the current study (65.2 U/mL/h). The relatively high production of fibrinolytic enzyme by the strain D21-8 indicated a promising prospect for industrial applications. It is noteworthy that the production cost for non-sterile fermentation of fibrinolytic enzyme by the strain D21-8 was reduced by 24% compared with that of sterile fermentation, and the sterilization cost constituted 17% of total fermentation cost in sterile fermentation. Zeng et al. (2013) first investigated co-production of poly(γ-glutamic acid) and fibrinolytic enzyme by a thermophilic *B. subtilis* GXA-28 at 45°C under non-sterile solid-state fermentation, which resulted in a fibrinolytic enzyme yield of 986 U/g-substrates and a relatively difficult process for enzyme purification owing to high viscosity caused by poly(γ-glutamic acid), thereby hindering industrial production of the fibrinolytic enzyme. The strain D21-8 employed in this study was the first reported to be capable of producing fibrinolytic enzyme under non-sterile submerged fermentation with high productivity and low operation cost.

On the other hand, YE and tryptone contributed to a large part of the production cost of fibrinolytic enzyme. Although the YE and tryptone used in this study were relatively expensive, they resulted in high production of fibrinolytic enzyme and important *in situ* antibacterial activity for contaminant control during non-sterile fermentation. The above results suggested the proposed non-sterile fermentation strategies were beneficial for improving fibrinolytic activity with low operation cost. Besides, it should be noted that the techo-economic assessments of the sterile and non-sterile processes in this study were based on the results of experiments at shake-flask level, which had limitations in evaluating fermentation benefits of fibrinolytic enzyme production in fermenters. However, this is the first report dealing with the non-sterile submerged fermentation of fibrinolytic enzyme and the relevant economic analysis, which could establish a platform for further scaling up of the non-sterile process in fermenters. Considering the economic aspect of the whole process, researchers are urged to develop low-cost media tailored to non-sterile submerged fermentation on the basis of elucidating fermentative characteristics of the strains as well as applying efficient strategies for contaminant control.

Furthermore, the purification of the fibrinolytic enzyme produced by *B. subtilis* D21-8 using the non-sterile optimized medium was workable according to our previous protocol for isolating the same enzyme from *B. subtilis* HQS-3 (Huang et al., 2013). The characterization of the antibacterial substances synthesized by the strain D21-8 is still ongoing. The commercially viable co-production and purification of the fibrinolytic enzyme and the antibacterial compounds under non-sterile conditions in the future would enable their applications as therapeutic agents.

## Conclusion

The starvation strategy by manipulating the medium composition helped in creating a growth-limiting condition for biological control. *In situ* antibacterial activity guaranteed a safe environment for fibrinolytic enzyme production. YE and tryptone were key requisites for efficient synthesis of fibrinolytic enzyme and antimicrobial compounds. The novel combination of starvation strategy and self-production of antimicrobials rendered higher fibrinolytic enzyme production with lower operation cost in shake-flask cultures under the non-sterile condition than under the conventional aseptic condition, providing valuable insights for developing efficient, cost-saving and robust processes for non-sterile fermentation of biochemicals.

## Author Contributions

All authors listed have made a substantial, direct and intellectual contribution to the work, and approved it for publication.

## Conflict of Interest Statement

The authors declare that the research was conducted in the absence of any commercial or financial relationships that could be construed as a potential conflict of interest.
